# Highlight report: Cardiotoxicity screening

**DOI:** 10.17179/excli2016-180

**Published:** 2016-02-22

**Authors:** Agapios Sachinidis

**Affiliations:** 1 Institute of Neurophysiology and Centre for Molecular Medicine Cologne (CMMC), Robert-Koch-Str. 39, 50931 Cologne, Germany

## ⁯

Recently, Bin Zhao and colleagues from the Chinese Academy of Science in Beijing and University of California, Sacramento, have published a review about identification of cardiotoxic compounds by rapid screening methods (Li et al., 2015[15]). Cardiotoxicity is one of the most frequent causes for withdrawal of drugs from the market and also during drug development (MacDonald and Robertson, 2009[16]; Apostolakis et al., 2013[1]; Li et al., 2015[15]). Totally, 81 drugs have been taken from the market between 1990 and 2013 (Li et al., 2015[15]). Other drugs, such as the antidiabetic rosiglitazone have been amended with cautionary notes informing about possible cardiotoxicity (Sager et al., 2015[18]). Therefore, it is of high relevance to establish screening methods for identification of cardiotoxic compounds and exclude them already during an early stage of drug development.

A relatively high number of withdrawn drugs have been shown to cause arrhythmia by blocking the hERG channel, a potassium channel responsible for repolarizing the cardiac action potential (Li et al., 2015[15]). The likelihood to block hERG increases with high lipophilicity, the presence of a positively charged nitrogen atom and the absence of negatively charged oxygen atoms (Villoutreix and Taboureau, 2015[22]). Meanwhile, software is available to identify possible interactions with hERG channel functions (reviewed in Li et al., 2015[15]). The authors recommend to first apply these in silico methods, followed by *in vitro* screening which also includes non-hERG drug targets. For this purpose, simple approaches, such as fluorescent imaging plate reader based assays and Ca^²+^ dye technologies represent convenient initial steps. Also, human pluripotent stem cells (hPSC) derived cardiomyocytes have been introduced and several gene and microRNA cardiotoxicity markers have been identified (Chaudhari et al., 2015[5], 2016[4]). Currently, large efforts are undertaken to establish alternative methods for toxicity testing, particularly in the fields of liver (Godoy et al., 2009[8], 2013[9], 2015[10]; Grinberg et al., 2014[12]), kidney (Yang et al., 2014[23]; Bulacio and Torres 2015[3]; Gong et al., 2015[11]) and neurotoxicity (Rempel et al., 2015[17]; Shinde et al., 2015[20]; Balmer et al., 2014[2]; Krug et al, 2013[14]), which are often supported by mathematical modeling (Drasdo et al., 2014[6]; Vartak et al., 2015[21]; Ghallab et al., 2015[7]; Schliess et al., 2014[19]; Hoehme et al., 2010[13]). The here discussed review of Li and colleagues gives a practical and helpful overview over currently available *in silio* and *in vitro* technologies for cardiotoxicity testing and critically discusses their possibilities as well as limitations.
